# Valve unit instead of intensive or intermediate care unit admission following transcatheter edge-to-edge mitral valve repair is safe and reduces postprocedural complications

**DOI:** 10.1007/s00392-024-02384-8

**Published:** 2024-02-14

**Authors:** Matthias Gröger, Dominik Felbel, Michael Paukovitsch, Leonhard Moritz Schneider, Sinisa Markovic, Wolfgang Rottbauer, Mirjam Keßler

**Affiliations:** Department of Internal Medicine II, Ulm University Heart Center, University of Ulm, Albert-Einstein-Allee 23, 89081 Ulm, Germany

**Keywords:** Mitral valve repair, TEER, Intensive care, Postprocedural care

## Abstract

**Background:**

Transcatheter edge-to-edge mitral valve repair (M-TEER) is often performed in general anesthesia, and postprocedural monitoring is usually warranted on an intensive or intermediate care unit (ICU/IMC). We evaluated the implications of a dedicated valve unit (VU) instead of an ICU/IMC for monitoring after M-TEER.

**Methods and results:**

In total, 624 patients were retrospectively analyzed. A total of 312 patients were primarily transferred to either ICU or IMC following M-TEER, and 312 patients were scheduled for the VU in the absence of indications for ICU/IMC treatment. Hospital stay was significantly shorter in VU patients (median 6.0 days (interquartile range (IQR) 5.0 – 8.0) vs. 7.0 days (IQR 6.0 – 10.0), *p* < 0.001) and their risk for infections (2.9 vs. 7.7%, *p* = 0.008) and delirium (0.6 vs. 2.6%, *p* = 0.056) was substantially lower compared to ICU/IMC patients. In-hospital mortality was similar in both groups (0.6% vs. 1.3%, *p* = 0.41).

Fifty patients (16.0%) in the VU group had to cross over to unplanned ICU/IMC admission. The most frequent indication was prolonged need for catecholamines (52.0%). Patients with ICU/IMC crossover had more advanced stages of heart failure (LV-EF < 30% in 36.0 vs. 16.0%, *p* = 0.001; severe concomitant tricuspid regurgitation in 48.0 vs. 27.8%, *p* = 0.005) and an LV-EF < 30% was independently associated with unplanned ICU/IMC admission.

**Conclusions:**

Following M-TEER postprocedural monitoring on a VU instead of an ICU/IMC is safe, reduces complications, and spares ICU capacities. Patients with advanced heart failure have a higher risk for unplanned ICU/IMC treatment after M-TEER.

**Graphical abstract:**

Valve unit instead of intensive or intermediate care unit admission following transcatheter edge-to-edge mitral valve repair is safe and reduces postprocedural complications.

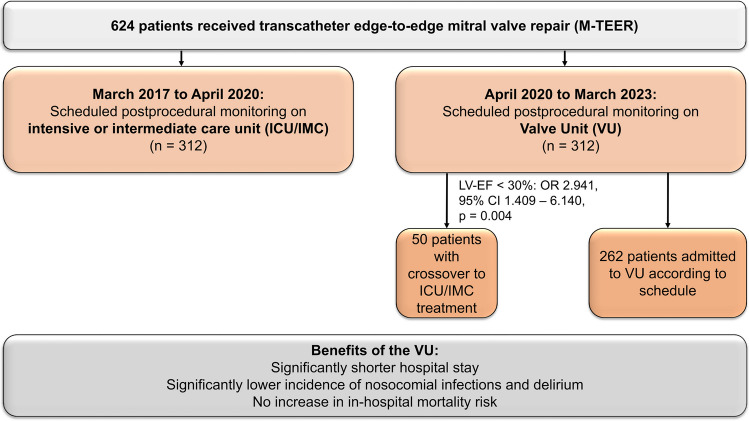

**Supplementary Information:**

The online version contains supplementary material available at 10.1007/s00392-024-02384-8.

## Introduction

Percutaneous transcatheter edge-to-edge mitral valve repair (M-TEER) is well established for interventional treatment of moderate to severe mitral regurgitation (MR) in symptomatic patients with high surgical risk and favorable anatomy [1]. Due to the need of periinterventional guidance by transesophageal echocardiography and for reasons of patient safety, the procedure is mostly performed in general anesthesia (GA) [2]. However, avoidance of GA and even of conscious sedation can be a feasible option for high-risk patients [3, 4]. The optimal postprocedural care for patients after M-TEER has not yet been defined. Intensive care unit (ICU) or intermediate care unit (IMC) admission can be seen as a standard due to putatively better response to complications [2, 5]. Such complications may include bleeding or infection [6, 7]. However, the COVID-19 pandemic has demonstrated the importance of careful utilization of intensive care capacities [8]. In response to the shortage of ICU and IMC resources during the pandemic, our center has established a dedicated valve unit (VU), spatially integrated into the general cardiology ward, for patient monitoring before and after valve procedures such as transfemoral aortic valve replacement (TAVR) or M-TEER in the absence of indications for ICU/IMC treatment. This study analyzes the feasibility and safety of a VU and its implications on patient outcomes and length of hospital stay.

## Methods

**Fig. 1:**
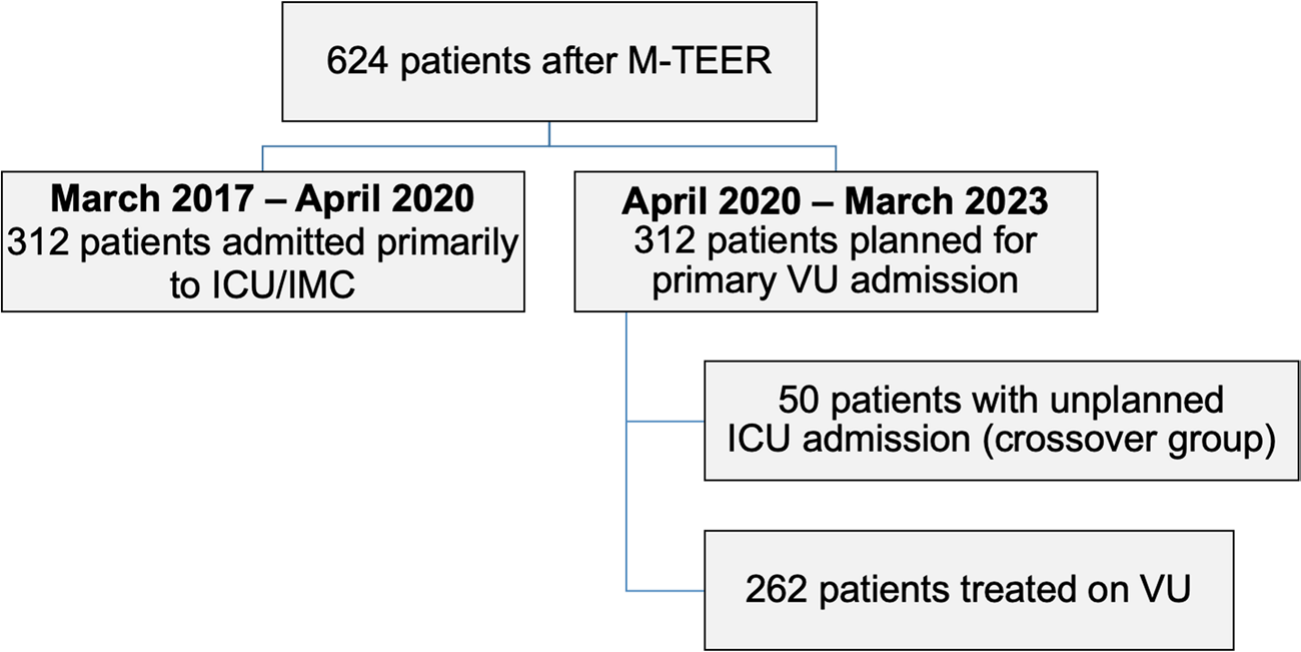
Overview of the study design. M-TEER, mitral valve transcatheter edge-to-edge repair; ICU, intensive care unit; IMC, intermediate care unit; VU, valve unit

We retrospectively examined 624 consecutive patients who underwent M-TEER in GA at our center from March 2017 to March 2023. Until April 2020, all 312 included patients were admitted to the ICU or IMC following the procedure. From April 2020, the following 312 patients were monitored on the valve unit (VU) immediately after the valve procedure in the absence of indications for ICU or IMC treatment. Patients, which were initially planned for VU admittance but required postprocedural ICU/IMC therapy (crossover group) were transferred to the VU after appropriate stabilization. The postprocedural course was registered until discharge. An overview of the study design is shown in Fig. 1.

All patients included in the present study were symptomatic in terms of heart failure (New York Heart Association (NYHA) functional class ≥ II) despite guideline-directed medical therapy. Echocardiographic characteristics at baseline and after the procedure were available for all study patients. Severity of MR was classified in four degrees according to the latest EACVI/ESC recommendations for MR quantification [9]. Left ventricular (LV) ejection fraction (LV-EF) was measured using the biplane Simpson method. LV diameters were measured in the parasternal long-axis view. Postprocedural MR severity was assessed by 2D and 3D transesophageal echocardiography after final device placement and removal of the guide catheter. MR was semi-quantitatively assessed by visual estimation of MR jet area and by (biplane) determination of the vena contracta of the major MR jet. In addition to MR severity, mitral valve gradients and area by 3D technique were assessed before and after device deployment and after removal of the guide catheter.

Device success was defined as TEER with reduction of MR of at least one degree with no more than moderate residual MR and absence of major device or procedure-related serious adverse events [10].

Standard for anti-infective prophylaxis was a single-shot of cefazolin before the start of the procedure. In case of known penicillin allergy, a single dose of levofloxacin was administered. Procedural duration was counted from the first femoral incision until access site closure. Time of anesthesia was counted from the start of induction of narcosis until extubation or, in case of prolonged ventilation, until handover to the ICU/IMC.

Statistical analysis was performed using SPSS 28 software (IBM Corp., Armonk, USA). Categorical variables are expressed as counts and percentages and were compared by Chi-square test or McNemar test, as appropriate. Continuous parameters were analyzed for normal distribution using Kolmogorov–Smirnov test. Normally distributed variables are presented as the mean ± standard deviation and were compared with *t*-test. Variables without normal distribution are shown as the median and quartiles and were compared with the Kruskal–Wallis test.

To determine predictors of unplanned ICU admission, univariate logistic regression analysis was performed for all potential influential variables (*p* < 0.2). In multivariate regression analysis, a backward stepwise algorithm was applied to all potentially influential parameters. Correlation was tested using Pearson’s correlation coefficient, Spearman’s rho, or Eta^2^ as appropriate. Variables with significant correlation were excluded from multivariate analysis. Due to the large number of covariates, stricter cutoffs for significant correlation were chosen (*p* < 0.05 or Eta^2^ > 0.2). Correlation analysis is shown in the Supplemental material.

All tests were two-tailed and differences were considered statistically significant when *p* < 0.05.

The study was ethically approved by the ethics committee of the University of Ulm (Approval Number 435/16) and complied with the principles outlined in the Declaration of Helsinki (Br Med J 1964; ii: 177).

## Results

### The valve unit

The VU was spatially integrated into a general cardiology ward at our tertiary care medical center. Patients were admitted to the VU before and immediately after valve procedures such as M-TEER, transcatheter edge-to-edge tricuspid valve repair (T-TEER), or TAVR in the absence of indications for ICU or IMC treatment. Invasive or non-invasive ventilation as well as administration of catecholamines was not to be performed on the VU. However, patient monitoring was intensified and followed a dedicated protocol. Monitoring consisted of continuous telemetric three-lead ECG recording and assessment of peripheral oxygen saturation as well as periodical non-invasive blood pressure measurements, all connected to a central alarm system. Compared to a standard cardiology ward, surveillance was intensified: intervals between blood pressure measurements were set to 10 min for the first hour after postprocedural admittance to the VU. Subsequently, half-hourly measurements were taken for 4 h. Under stable conditions, blood pressure measurements and telemetric monitoring were terminated after 4 h. Checkups by nursing staff were carried out quarter-hourly in the first hour after M-TEER followed by visits at least every 2 h until the following day. A physician was available 24 h and all medical staff were trained for specific aspects of care following GA and valve interventions, specifically including access site bleeding and other vascular complications, pericardial effusion, renal failure, or neurologic events. Nursing staff recorded all measurements and clinical events on dedicated VU patient files. In case of an uneventful postprocedural course, patients were discharged directly from the VU.

### Baseline characteristics and procedural details of the study subgroups

In total, 624 patients were analyzed consecutively. A total of 312 patients were transferred to the ICU/IMC following M-TEER, and 312 patients were scheduled for the VU. Apart from a lower prevalence of atrial fibrillation, there was no significant difference in demographics and comorbidities. Patients in the ICU/IMC era had larger left ventricles and more severe MR at baseline. Duration of the procedure and of anesthesia was significantly longer in these patients and device success was inferior compared to the VU era. Severe periinterventional complications were very rare and occurred equally in both groups. While in the ICU/IMC era the MitraClip platform (Abbott Cardiovascular) was used predominantly, procedures were performed in equal shares with both MitraClip and PASCAL (Edwards Lifesciences) platforms in the VU era. Median time spent on the ICU was 4.0 h in the ICU/IMC group while it was 0.0 h in the VU group. VU patients had a significantly shorter hospital stay.

Baseline characteristics and procedural details are shown in Table 1.

**Table 1:** Baseline characteristics and procedural details of patients admitted to the ICU and to the VU

Variable	ICU/IMC (*n* = 312)	VU (*n* = 312)	*p*
Baseline characteristics			
Age (years)	79.0 (74.0 – 82.0)	80.0 (74.0 – 84.0)	0.18
Female sex	134 (42.9%)	146 (46.8%)	0.33
Baseline NYHA Class			
II III IV	54 (17.3%) 194 (62.2%) 64 (20.5%)	54 (17.3%) 213 (68.3%) 45 (14.4%)	0.12
LV-EF			
≥ 50% 41 – 49% 30 – 40% < 30%	133 (42.6%) 53 (17.0%) 70 (22.4%) 56 (17.9%)	134 (42.9%) 67 (21.5%) 51 (16.3%) 60 (19.2%)	0.19
Severe concomitant TR	80 (26.6%)	96 (31.1%)	0.22
CAD	198 (63.5%)	190 (60.9%)	0.51
DCM	52 (16.7%)	65 (20.8%)	0.26
** Atrial fibrillation**	**179 (57.4%)**	**222 (71.2%)**	** < 0.001**
Obstructive lung disease	32 (10.3%)	30 (9.6%)	0.79
Body mass index (kg/m^2^)	25.4 (22.8 – 28.1)	25.7 (23.0 – 28.9)	0.27
Creatinine (µmol/l)	114.0 (90.0 – 147.0)	110.0 (87.0 – 143.0)	0.26
eGFR (ml/min)	48.0 (34.0 – 61.8)	48.0 (34.0 – 66.0)	0.57
Hemoglobine (g/dl)	12.7 (11.4 – 13.9)	12.9 (11.4 – 13.9)	0.43
Troponin T (ng/l)	28.0 (18.0 – 44.5)	26.0 (17.0 – 45.0)	0.87
NT-proBNP (pg/ml)	2770.0 (1282.0 – 6002.0)	2397.0 (1049.3 – 4872.3)	0.07
**LVEDD (mm)**	**59.0 (52.0 – 67.0)**	**57.0 (51.0 – 64.0)**	**0.047**
EuroSCORE II	4.7 (2.6 – 8.3)	4.9 (3.1 – 8.6)	0.143
STS-Score	3.2 (2.0 – 6.8)	4.1 (2.2 – 7.0)	0.06
sPAP (mmHg)	50.4 ± 16.0	51.8 ± 14.1	0.49
mPAP (mmHg)	32.5 ± 10.1	32.7 ± 8.9	0.84
MR etiology			
Degenerative Functional Mixed	124 (39.7%) 124 (39.7%) 64 (20.5%)	109 (34.9%) 147 (47.1%) 56 (17.9%)	0.18
**Baseline MR grade**			
** III** **IV**	**68 (21.8%)** **244 (78.2%)**	**92 (29.5%)** **220 (70.5%)**	**0.04**
Procedural details			
** Device success**	**279 (89.4%)**	**299 (95.8%)**	**0.002**
** Platform used**			
**MitraClip** **PASCAL**	**293 (93.9%)** **19 (6.1%)**	**138 (44.2%)** **174 (55.8%)**	** < 0.001**
** Procedural duration (min)**	**84.0 (66.0 – 107.5)**	**79 (61.3 – 100.0)**	**0.011**
**Anesthesia duration (min)**	**120.0 (105.0 – 150.0)**	**120 (95.0 – 138.8)**	** < 0.001**
**Postprocedural MR grade**			
** < I** ** I** ** II** ** III** **IV**	**47 (15.1%)** **128 (41.0%)** **109 (34.9%)** **28 (9.0%)** **0**	**90 (28.8%)** **141 (45.2%)** **69 (22.1%)** **11 (3.5%)** **1 (0.3%)**	** < 0.001**
Postprocedural MV-PGmean (mmHg)	3.0 (2.0 – 5.0)	3.0 (2.0 – 4.0)	0.24
SLDA	1 (0.3%)	1 (0.3%)	0.99
Clip embolization	0	1 (0.3%)	0.32
Periinterventional cardiogenic shock	2 (0.6%)	3 (1.0%)	0.65
Periinterventional CPR	6 (1.9%)	2 (0.6%)	0.16
Periinterventional stroke	1 (0.3%)	0	0.32
** Time on ICU/IMC (h)**	**4.0 (3.0 – 9.0)**	**0.0 (0.0 – 0.0)**	** < 0.001**
** Time in hospital (days)**	**7.0 (6.0 – 10.0)**	**6.0 (5.0 – 8.0)**	** < 0.001**

Statistical significance marked in bold (*p* < 0.05)

*ICU* intensive care unit, *VU* valve unit, *NYHA* New York Heart Association, *LV-EF* left ventricular ejection fraction, *TR* tricuspid regurgitation, *CAD* coronary artery disease, *DCM* dilatative cardiomyopathy, *eGFR* estimated glomerular filtration rate, *LVEDD* left ventricular end-diastolic diameter, *STS* Society of Cardiothoracic Surgeons, *sPAP* systolic pulmonary artery pressure, *mPAP* mean pulmonary artery pressure, *MR* mitral regurgitation, *MV-PGmean* mean transmitral pressure gradient, *SLDA* single leaflet device attachment, *CPR* cardiopulmonary resuscitation. Statistical significance marked in bold (p < 0.05).

### Postprocedural complication rates in the ICU and the VU era

**Fig. 2:**
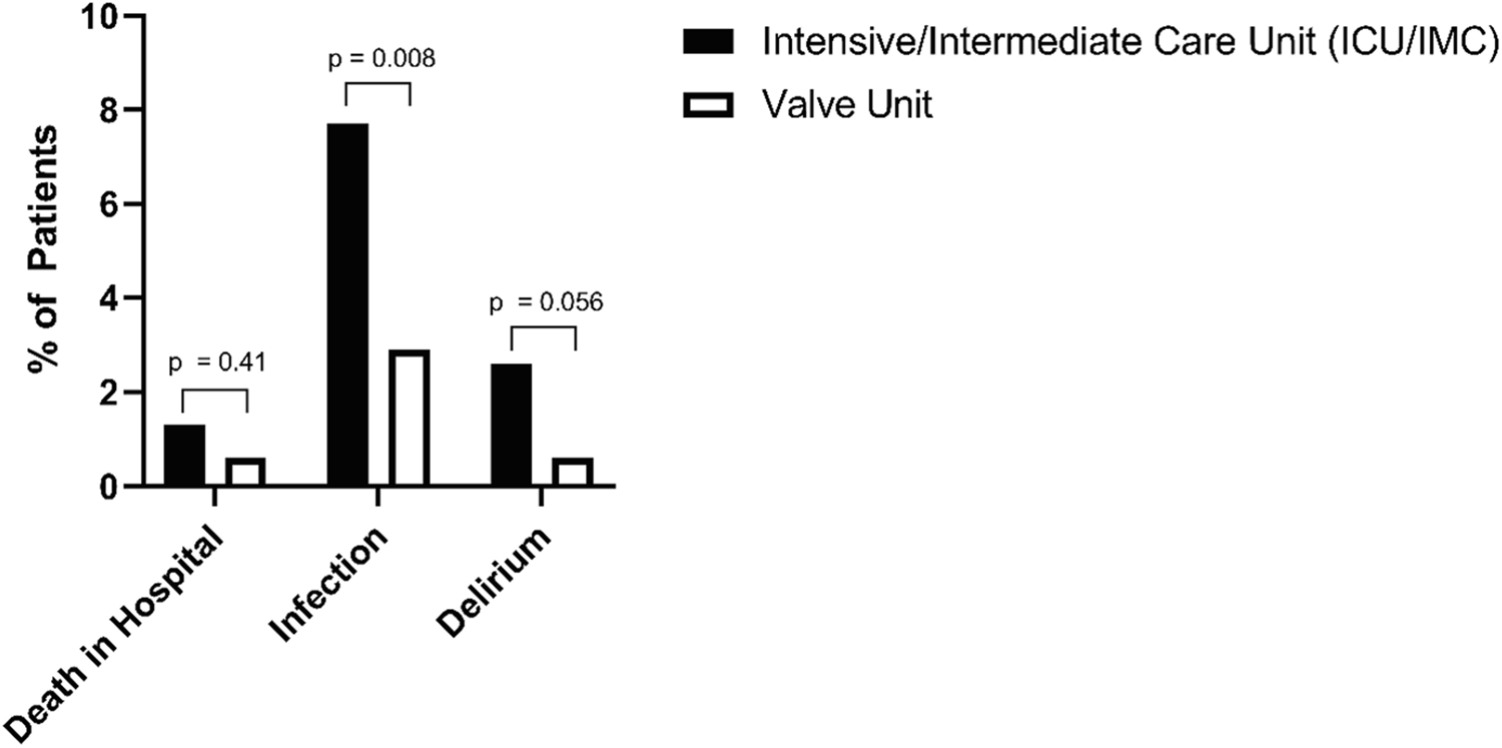
Incidence of postprocedural complications after M-TEER in the ICU/IMC and the VU groups. M-TEER, mitral valve transcatheter edge-to-edge repair; ICU, intensive care unit; IMC, intermediate care unit; VU, valve unit

In an “intention-to-treat” analysis, the incidence of infections was significantly lower in the VU era compared to the ICU/IMC group (2.9 vs. 7.7%, *p* = 0.008). The incidence of postprocedural delirium was also considerably lower (0.6 vs. 2.6%, *p* = 0.056). In-hospital mortality overall was very rare and was similar in both groups (0.6% in the VU group, 1.3% in the ICU group, *p* = 0.41) (Fig. 2). These effects were even more pronounced in an “as-treated” analysis combining planned ICU/IMC admissions and crossovers: infections occurred in 7.2% of patients that were treated on the ICU/IMC compared to 2.7% of patients that had avoided the ICU (*p* = 0.013). Incidences of postprocedural delirium (2.8 vs. 0%, *p* = 0.007) and in-hospital mortality (1.7 vs. 0%, *p* = 0.037) were also significantly higher in these patients. Furthermore, duration of the procedure or anesthesia was similar in patients with and without development of infection or delirium (median procedural duration: 75.0 (65.0 – 112.0) vs. 83.0 min (63.0 – 103.0), *p* = 0.53; median anesthesia duration: 120.0 (105.0 – 165.0) vs. 120.0 min (100.0 – 145.0), *p* = 0.57).

### Crossover to unplanned ICU admission in the VU era

**Fig. 3:**
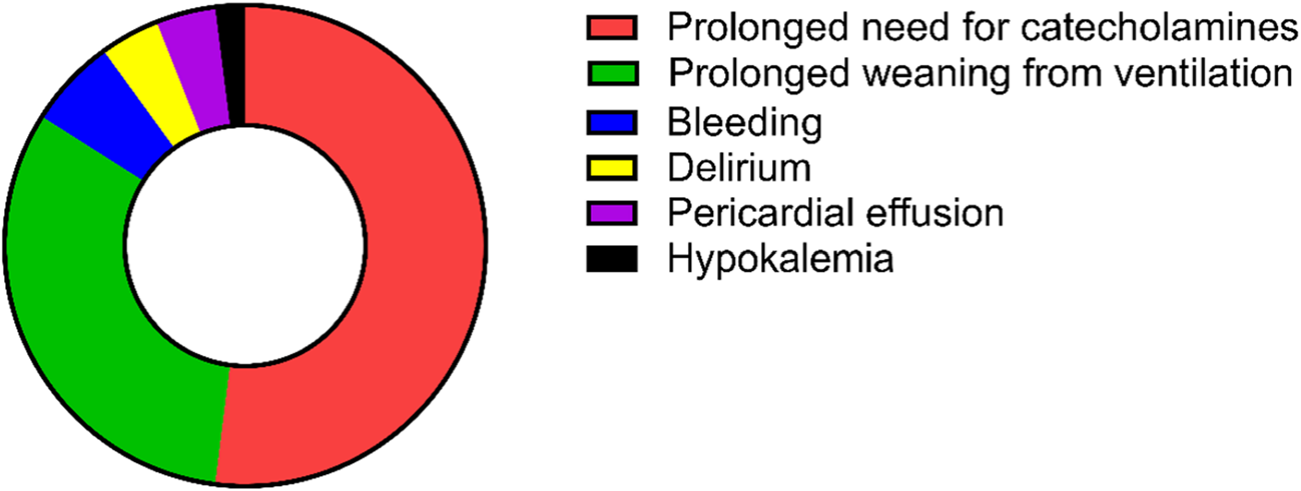
Indications for crossover to unplanned ICU admission after M-TEER. M-TEER, mitral valve transcatheter edge-to-edge repair; ICU, intensive care unit

Fifty patients (16.0%) that were scheduled to be relocated to the VU following M-TEER instead had to be transferred to the ICU/IMC (crossover group). The most frequent indication for ICU/IMC treatment in these patients was requirement of catecholamines (52.0%) followed by prolonged ventilation (32.0%) (Fig. 3). Patients in the crossover group (see Table 2) were younger and more often male and had significantly more pronounced heart failure signs: higher baseline New York Heart Association (NYHA) functional class, lower LV-EF, higher prevalence of dilatative cardiomyopathy, and severe concomitant tricuspid regurgitation (TR). Their LV diameters were significantly larger, and Troponin T and NT-proBNP significantly higher. A significantly higher EuroSCORE II reflected the higher overall morbidity. Furthermore, procedural duration was significantly longer in these patients. There was no relevant difference in pre-procedural MR grade or device success rate. Median duration of the unscheduled ICU stay was 20.0 h (IQR 5.0 – 29.3 h). Two patients died while in hospital (4.0%).

**Table 2:** Baseline characteristics and procedural details of patients with and without unplanned ICU admission in the VU era

Variable	No ICU/IMC admission (*n* = 262)	Unplanned ICU/IMC admission (*n* = 50)	*p*
Baseline characteristics			
** Age (years)**	**81.0 (74.0 – 84.0)**	**76.5 (68.0 – 83.0)**	**0.009**
** Female sex**	**129 (49.2%)**	**17 (34.0%)**	**0.048**
** Baseline NYHA Class**			
**II** **III** **IV**	**52 (19.8%)** **177 (67.6%)** **33 (12.6%)**	**2 (4.0%)** **36 (72.0%)** **12 (24.0%)**	**0.007**
** LV-EF**			
**≥ 50%** **41 – 49%** ** 30 – 40%** ** < 30%**	**121 (46.2%)** **59 (22.5%)** **40 (15.3%)** **42 (16.0%)**	**13 (26.0%)** **8 (16.0%)** **11 (22.0%)** **18 (36.0%)**	**0.002**
** Severe concomitant TR**	**72 (27.8%)**	**24 (48.0%)**	**0.005**
CAD	160 (61.1%)	30 (60.0%)	0.89
** DCM**	**49 (18.7%)**	**16 (32.0%)**	**0.03**
Atrial fibrillation	185 (70.6%)	37 (74.0%)	0.63
Obstructive lung disease	26 (9.9%)	4 (8.0%)	0.67
Body mass index (kg/m^2^)	25.7 (22.9 – 28.6)	26.2 (23.1 – 30.0)	0.23
Creatinine (µmol/l)	106.0 (86.0 – 139.0)	120.0 (97.0 – 159.3)	0.06
eGFR (ml/min)	49.0 (33.5 – 67.5)	43.5 (34.5 – 62.3)	0.38
Hemoglobine (g/dl)	12.9 (11.5 – 14.0)	12.8 (10.5 – 13.3)	0.11
** Troponin T (ng/l)**	**25.0 (17.0 – 43.0)**	**37.5 (23.5 – 70.3)**	**0.005**
** NT-proBNP (pg/ml)**	**2140.0 (938.0 – 4011.0)**	**4671.0 (1745.0 – 8887.0)**	** < 0.001**
** LVEDD (mm)**	**56.0 (50.0 – 63.0)**	**62.0 (52.0 – 71.3)**	**0.011**
** EuroSCORE II**	**4.8 (2.9 – 8.3)**	**7.3 (3.8 – 10.8)**	**0.021**
STS score	4.0 (2.1 – 7.0)	4.3 (2.6 – 12.0)	0.27
sPAP (mmHg)	51.1 ± 14.1	57.9 ± 13.2	0.09
** mPAP (mmHg)**	**32.1 ± 8.6**	**38.5 ± 9.8**	**0.033**
MR etiology			
Degenerative Functional Mixed	97 (37.0%) 118 (45.0%) 47 (17.9%)	12 (24.0%) 29 (58.0%) 9 (18.0%)	0.17
Baseline MR grade			
III IV	82 (31.3%) 180 (68.7%)	10 (20.0%) 40 (80.0%)	0.11
Procedural details			
Device success	252 (96.2%)	47 (94.0%)	0.48
Device used			
MitraClip PASCAL	118 (45.0%) 144 (55.0%)	20 (40.0%) 30 (60.0%)	0.51
** Procedural duration (min)**	**77.0 (60.8 – 99.0)**	**85.5 (65.0 – 113.0)**	**0.03**
Anesthesia duration (min)	120.0 (95.0 – 135.0)	120 (100.0 – 151.3)	0.051
Postprocedural MR grade			
< I	73 (27.9%)	17 (34.0%)	
I	125 (47.7%)	16 (32.0%)	
II	55 (21.0%)	14 (28.0%)	
III	8 (3.1%)	3 (6.0%)	0.29
IV	1 (0.4%)	0	
Postprocedural MV-PGmean (mmHg)	3.0 (2.0 – 4.0)	3.0 (2.0 – 5.0)	0.80
SLDA	1 (0.3%)	0	0.66
Clip embolization	1 (0.3%)	0	0.66
** Periinterventional cardiogenic shock**	**0**	**3 (6.0%)**	** < 0.001**
** Periinterventional CPR**	**0**	**2 (4.0%)**	**0.001**
Periinterventional stroke	0	0	1.0
** Time on ICU/IMC (h)**	**0.0 (0.0 – 0.0)**	**20.0 (5.0 – 29.3)**	** < 0.001**
** Time in hospital (days)**	**6.0 (5.0 – 7.0)**	**7.0 (6.0 – 11.0)**	** < 0.001**

Statistical significance marked in bold (*p* < 0.05)

*ICU* intensive care unit, *VU* valve unit, *NYHA* New York Heart Association, *LV-EF* left ventricular ejection fraction, *TR* tricuspid regurgitation, *CAD* coronary artery disease, *DCM* dilatative cardiomyopathy, *eGFR* estimated glomerular filtration rate, *LVEDD* left ventricular end-diastolic diameter, *STS* Society of Cardiothoracic Surgeons, *sPAP* systolic pulmonary artery pressure, *mPAP* mean pulmonary artery pressure, *MR* mitral regurgitation, *MV-PGmean* mean transmitral pressure gradient, *SLDA* single leaflet device attachment, *CPR* cardiopulmonary resuscitation.

Multivariate logistic regression analysis identified an LV-EF of < 30% as an independent predictor of unscheduled ICU/IMC admission (hazard ratio 2.941, 95% confidence interval 1.409 – 6.140, *p* = 0.004, Table 3).

**Table 3:** Univariate and multivariate Log regression to identify predictors of unscheduled ICU admission following M-TEER

	Univariate Log regression analysis			Multivariate Log regression analysis		
	Odds ratio	95% confidence interval	*p*	Odds ratio	95% confidence interval	*p*
Age	0.952	0.921 – 0.984	0.003			
Female sex	0.531	0.282 – 1.001	0.050			
Baseline NYHA class IV	2.191	1.041 – 4.614	0.039	1.743	0.761 – 3.992	0.189
**LV-EF < 30%**	**3.031**	**1.632 – 5.631**	** < 0.001**	**2.941**	**1.409 – 6.140**	**0.004**
Severe concomitant TR	2.397	1.292 – 4.447	0.006			
DCM	2.046	1.046 – 3.999	0.036			
Creatinine	1.002	0.998 – 1.006	0.272			
Hb	0.883	0.757 – 1.029	0.111			
Troponin T	1.002	1.000 – 1.005	0.045			
NT-proBNP (per 1.000 pg/ml)	1.003	0.989 – 1.016	0.713			
LVEDD	1.040	1.006 – 1.075	0.020			
FMR	1.685	0.914 – 3.108	0.095			
Baseline MR grade IV	1.822	0.869 – 3.821	0.112	1.575	0.690 – 3.592	0.280
Procedural duration	1.006	0.999 – 1.013	0.078	1.005	0.998 – 1.012	0.130

Statistical significance marked in bold (*p* < 0.05)

*M-TEER* mitral valve transcatheter edge-to-edge repair, *NYHA* New York Heart Association, *LV-EF* left ventricular ejection fraction, *TR* tricuspid regurgitation, *DCM* dilatative cardiomyopathy, *Hb* hemoglobin, *LVEDD* left ventricular end-diastolic diameter, *FMR* functional mitral regurgitation, *MR* mitral regurgitation.

## Discussion

With the growing importance of M-TEER and its establishment as a routine intervention in experienced heart centers, a standardization of postprocedural care is necessary. Routine ICU monitoring after M-TEER has been suggested and is commonly performed, at least after procedures in GA [2]. However, ICU and IMC capacities are a valuable resource and clinicians might encounter the dilemma of maintaining optimal patient safety on the one hand and mindful use of ICU and IMC structures as well as cost-effectiveness on the other. Our data show that implementation of a dedicated VU for post-interventional monitoring is a reasonable option.

Immediate admission to the VU instead of primary ICU/IMC treatment after M-TEER was associated with a significantly shorter hospital stay and a lower incidence of infections and delirium. Importantly, these complications occurred irrespective of procedural duration or duration of anesthesia. Both adverse events are known to occur more frequently in patients treated in an ICU [11, 12]. In a study by Körber et al., periinterventional delirium was associated with longer hospital stay and higher risk for bleeding and periprocedural infections in patients undergoing M-TEER or T-TEER [13]. In our study, avoidance of ICU/IMC admission has certainly streamlined the postprocedural course and therefore shortened the time spent in hospital and the exposure to risk factors for infections and delirium. However, longer procedural and anesthesia duration and inferior procedural success rates in the ICU/IMC era might have prolonged hospital stay in this group.

Most importantly, in-hospital mortality in our study was non-inferior in VU patients compared to the ICU/IMC group. Thus, a step down in postprocedural surveillance does not come at the prize of impaired patient safety.

To date, the adequate postprocedural monitoring of M-TEER patients has not been studied well. Efforts to streamline interventional and periinterventional processes have mainly been made in TAVR patients [14–16]. In the context of M-TEER, data by Di Prima et al. have shown that while all patients in their study were admitted to an ICU after the intervention, 18.5% left the ICU on the same day due to an uneventful postprocedural course and 68.5% could be transferred to a general ward the day after [5]. Our data now show for the first time that routine ICU/IMC treatment following M-TEER in GA is not required in the majority of patients. In fact, only 16.0% of patients had to be admitted to the ICU/IMC while 84.0% received adequate postprocedural care on the VU. According to our data, however, patients with reduced LV-EF should be monitored closely as they have a significantly higher risk to require ICU or IMC treatment compared to patients with better systolic function.

Notably, the most frequent causes for unscheduled ICU/IMC transfer after M-TEER in our cohort were prolonged need for catecholamines and protracted weaning from mechanical ventilation. Both factors are closely related to GA. However, avoidance of GA and mechanical ventilation has been shown to be a feasible option especially for patients at higher risk for anesthesia-associated complications [17, 18]. In a meta-analysis, Banga et al. reported a shorter ICU stay and a comparable rate of procedural success by using conscious or deep sedation compared to GA [19]. As crossover to ICU/IMC treatment occurred significantly more frequently in patients with advanced heart failure, preferring sedation to GA might be a feasible option to further reduce ICU admissions in these high-risk patients. Though our study cannot report on the course of patients receiving M-TEER using deep sedation only, an additional benefit of ICU/IMC avoidance in these patients is conceivable.

The main differences in patient characteristics in the ICU/IMC and the VU era were procedure-related. Lower device success rates and longer duration of the procedure and anesthesia might have influenced outcomes. The superior procedural results in the VU era are likely due to substantial technical advances of TEER devices which have improved TEER results in general [20]. Significant differences in the choice of the TEER device are owed to the later market introduction of the PASCAL device, which was not yet used broadly in our center in the ICU/IMC era.

### Study limitations

Our data resemble a retrospective study and while we report on a substantially sized cohort our findings are limited to a single-center experience. Further external evaluation of strategies of ICU/IMC avoidance after M-TEER should be sought to validate our conclusions. Secondly, a potential bias may arise from the long period of time included in our analysis and the respective technical and procedural advancements in this era. Furthermore, certain important complications such as bleeding have not been routinely registered throughout the observation period and are therefore not included in this study.

## Conclusions

Implementation of a VU for postprocedural monitoring after M-TEER instead of primary routine ICU/IMC admission maintains patient safety, reduces complications, and may even reduce hospital stay. Patients with advanced heart failure have a higher risk of unplanned crossover to ICU/IMC treatment and an LV-EF of < 30% is an independent predictor of ICU/IMC admission. Use of a VU as a standardized target structure for M-TEER patients may help to streamline the procedure, reduce healthcare costs, and spare ICU capacities.

## Supplementary Information

Below is the link to the electronic supplementary material.

Supplementary file1 (DOCX 17 KB)

## Data Availability

The data underlying this article will be shared on reasonable request to the corresponding author.
