# Supplementary material for: Valve unit instead of intensive or intermediate care unit admission following transcatheter edge-to-edge mitral valve repair is safe and reduces postprocedural complications
Source: Clin Res Cardiol. 2024 Feb 14;114(7):818–26. doi: 10.1007/s00392-024-02384-8 (PMC12202642; doi:10.1007/s00392-024-02384-8)
Supplement: Supplementary file 1 — Supplementary file1 (DOCX 17 KB) [file 392_2024_2384_MOESM1_ESM.docx]

**Supplemental Material:**

Correlation analysis of factors potentially associated with unplanned crossover to ICU treatment

|  | Age | Female Sex | Baseline NYHA Class IV | LV-EF < 30% | Severe Concomitant TR | DCM | Creatinine | Hb | Troponin T | NT-proBNP | LVEDD | FMR | Baseline MR Grade IV | Procedural Duration |
| --- | --- | --- | --- | --- | --- | --- | --- | --- | --- | --- | --- | --- | --- | --- |
| Age | - | η = 0.109 | η = 0.050 | η = 0.131 | η = 0.173 | η = 0.146 | **ρ = 0.162**  **p = 0.004** | **ρ = -0.192**  **p < 0.001** | **ρ = 0.239**  **p < 0.001** | **ρ = 0.182**  **p = 0.002** | **ρ = -0.172**  **p =0.010** | η = 0.100 | η = 0.038 | ρ = -0.077  p = 0.202 |
| Female Sex | η = 0.109 | - | φ = -0.021  p = 0.716 | **φ = -0.164**  **p = 0.004** | φ = 0.100  p = 0.078 | φ = -0.070  p = 0.217 | η = 0.185 | η = 0.184 | η = 0.109 | η = 0.072 | η = 0.323 | φ = -0.010  p = 0.858 | **φ = -0.112**  **p = 0.048** | η = 0.032 |
| Baseline  NYHA Class IV | η = 0.050 | φ = -0.021  p = 0.716 | - | φ = 0.057  p = 0.311 | φ = 0.056  p = 0.326 | φ = 0.039  p = 0.488 | η = 0.090 | η = 0.089 | η = 0.184 | **η = 0.220** | η = 0.005 | φ = 0.034  p = 0.545 | φ = 0.086  p = 0.128 | η = 0.179 |
| LV-EF  < 30% | η = 0.131 | **φ = -0.164**  **p = 0.004** | φ = 0.057  p = 0.311 | - | **φ = 0.133**  **p = 0.020** | **φ = 0.210**  **p < 0.001** | η = 0.086 | η = 0.064 | η = 0.099 | η = 0.063 | **η = 0.354** | **φ = 0.354**  **p < 0.001** | φ = -0.023  p = 0.680 | η = 0.010 |
| Severe Concomitant TR | η = 0.173 | φ = 0.100  p = 0.078 | φ = 0.056  p = 0.326 | **φ = 0.133**  **p = 0.020** | - | φ = 0.062  p = 0.280 | η = 0.024 | **η = 0.207** | η = 0.032 | η = 0.126 | η = 0.071 | φ = 0.058  p = 0.306 | φ = -0.068  p = 0.233 | η = 0.148 |
| DCM | η = 0.146 | φ = -0.070  p = 0.217 | φ = 0.039  p = 0.488 | **φ = 0.210**  **p < 0.001** | φ = 0.062  p = 0.280 | - | η = 0.115 | η = 0.093 | η = 0.039 | η = 0.029 | **η = 0.364** | **φ = 0.227**  **p < 0.001** | φ = -0.032  p = 0.575 | η = 0.005 |
| Creatinine | **ρ = 0.162**  **p = 0.004** | η = 0.185 | η = 0.090 | η = 0.086 | η = 0.024 | η = 0.115 | - | **ρ = -0.270**  **p < 0.001** | **ρ = 0.602**  **p < 0.001** | **ρ = 0.511**  **p < 0.001** | **ρ = 0.148**  **p = 0.028** | η = 0.197 | η = 0.077 | ρ = -0.023  p = 0.709 |
| Hb | **ρ = -0.192**  **p < 0.001** | η = 0.184 | η = 0.089 | η = 0.064 | **η = 0.207** | η = 0.093 | **ρ = -0.270**  **p < 0.001** | - | **ρ = -0.307**  **p < 0.001** | **ρ = -0.270**  **p < 0.001** | ρ = 0.020  p = 0.768 | η = 0.101 | η = 0.047 | ρ = 0.017  p = 0.775 |
| Troponin T | **ρ = 0.239**  **p < 0.001** | η = 0.109 | η = 0.184 | η = 0.099 | η = 0.032 | η = 0.039 | **ρ = 0.602**  **p < 0.001** | **ρ = -0.307**  **p < 0.001** | - | **ρ = 0.607**  **p < 0.001** | **ρ = 0.143**  **p = 0.035** | η = 0.151 | η = 0.061 | ρ = -0.004  p = 0.953 |
| NT-proBNP  (per 1.000 pg/ml) | **ρ = 0.182**  **p = 0.002** | η = 0.072 | η = 0.220 | η = 0.063 | η = 0.126 | η = 0.029 | **ρ = 0.511**  **p < 0.001** | **ρ = -0.270**  **p < 0.001** | **ρ = 0.607**  **p < 0.001** | - | **ρ = 0.217**  **p = 0.001** | η = 0.019 | η = 0.041 | ρ = 0.105  p = 0.090 |
| LVEDD | **ρ = -0.172**  **p =0.010** | **η = 0.323** | η = 0.005 | **η = 0.354** | η = 0.071 | **η = 0.364** | **ρ = 0.148**  **p = 0.028** | ρ = 0.020  p = 0.768 | **ρ = 0.143**  **p = 0.035** | **ρ = 0.217**  **p = 0.001** | - | **η = 0.288** | η = 0.032 | ρ = 0.101  p = 0.156 |
| FMR | η = 0.100 | φ = -0.010  p = 0.858 | φ = 0.034  p = 0.545 | **φ = 0.354**  **p < 0.001** | φ = 0.058  p = 0.306 | **φ = 0.227**  **p < 0.001** | η = 0.197 | η = 0.101 | η = 0.151 | η = 0.019 | **η = 0.288** | - | **φ = -0.178**  **p = 0.002** | η = 0.002 |
| Baseline  MR Grade IV | η = 0.038 | **φ = -0.112**  **p = 0.048** | φ = 0.086  p = 0.128 | φ = -0.023  p = 0.680 | φ = -0.068  p = 0.233 | φ = -0.032  p = 0.575 | η = 0.077 | η = 0.047 | η = 0.061 | η = 0.041 | η = 0.032 | **φ = -0.178**  **p = 0.002** | - | η = 0.185 |
| Procedural Duration | ρ = -0.077  p = 0.202 | η = 0.032 | η = 0.179 | η = 0.010 | η = 0.148 | η = 0.005 | ρ = -0.023  p = 0.709 | ρ = 0.017  p = 0.775 | ρ = -0.004  p = 0.953 | ρ = 0.105  p = 0.090 | ρ = 0.101  p = 0.156 | η = 0.002 | η = 0.185 | - |

*NYHA: New York Heart Association; LV-EF: left-ventricular ejection fraction; TR: tricuspid regurgitation; DCM: dilatative cardiomyopathy; Hb: Hemoglobine; LVEDD: left-ventricular end-diastolic diameter; FMR: functional mitral regurgitation; MR: mitral regurgitation*
